# Adult Multipotent Cardiac Progenitor-Derived Spheroids: A Reproducible Model of In Vitro Cardiomyocyte Commitment and Specification

**DOI:** 10.3390/cells12131793

**Published:** 2023-07-05

**Authors:** Mariangela Scalise, Fabiola Marino, Luca Salerno, Nunzia Amato, Claudia Quercia, Chiara Siracusa, Andrea Filardo, Antonio Chiefalo, Loredana Pagano, Giuseppe Misdea, Nadia Salerno, Antonella De Angelis, Konrad Urbanek, Giuseppe Viglietto, Daniele Torella, Eleonora Cianflone

**Affiliations:** 1 Department of Experimental and Clinical Medicine, Magna Graecia University, 88100 Catanzaro, Italy; m.scalise@unicz.it (M.S.); marino@unicz.it (F.M.); l.salerno@unicz.it (L.S.); antonio.chiefalo@unicz.it (A.C.); giuseppe.misdea002@studenti.unicz.it (G.M.); nadia.salerno@unicz.it (N.S.); viglietto@unicz.it (G.V.); 2 Department of Medical and Surgical Sciences, Magna Graecia University, 88100 Catanzaro, Italy; nunzia.amato@studenti.unicz.it (N.A.); claudia.quercia@unicz.it (C.Q.); chiara.siracusa@unicz.it (C.S.); filardo@unicz.it (A.F.); loredana.pagano@studenti.unicz.it (L.P.); 3 Department of Experimental Medicine, University of Campania “L. Vanvitelli”, 80138 Naples, Italy; antonella.deangelis@unicampania.it; 4 Department of Molecular Medicine and Medical Biotechnology, Federico II University, 88121 Naples, Italy; konradarkadiusz.urbanek@unina.it

**Keywords:** 3D cell culture, cardiac spheroids, multipotent cardiac progenitors, cardiomyocyte differentiation

## Abstract

Background: Three-dimensional cell culture systems hold great promise for bridging the gap between in vitro cell-based model systems and small animal models to study tissue biology and disease. Among 3D cell culture systems, stem-cell-derived spheroids have attracted significant interest as a strategy to better mimic in vivo conditions. Cardiac stem cell/progenitor (CSC)-derived spheroids (CSs) provide a relevant platform for cardiac regeneration. Methods: We compared three different cell culture scaffold-free systems, (i) ultra-low attachment plates, (ii) hanging drops (both requiring a 2D/3D switch), and (iii) agarose micro-molds (entirely 3D), for CSC-derived CS formation and their cardiomyocyte commitment in vitro. Results: The switch from a 2D to a 3D culture microenvironment per se guides cell plasticity and myogenic differentiation within CS and is necessary for robust cardiomyocyte differentiation. On the contrary, 2D monolayer CSC cultures show a significant reduced cardiomyocyte differentiation potential compared to 3D CS culture. Forced aggregation into spheroids using hanging drop improves CS myogenic differentiation when compared to ultra-low attachment plates. Performing CS formation and myogenic differentiation exclusively in 3D culture using agarose micro-molds maximizes the cardiomyocyte yield. Conclusions: A 3D culture system instructs CS myogenic differentiation, thus representing a valid model that can be used to study adult cardiac regenerative biology.

## 1. Introduction

Three-dimensional (3D) cell culture systems hold great promise for bridging the gap between in vitro cell-based model systems and small animal models to study tissue/organ biology, development, and disease, to investigate novel drugs and therapeutics and to find new effective strategies for the repair and replacement of tissues and organs [1,2]. Three-dimensional cell culture systems have indeed attracted significant interest as a strategy to better mimic in vivo conditions, such as the complex cell–cell and cell–cell–extra cellular matrix (ECM) interactions [1]. Three-dimensional cell cultures, such as spheroids, guide the specific functions and growth/differentiation of cells (e.g., embryogenesis, morphogenesis, and organogenesis). Thus, 3D cell culture is a promising in vitro tool to generate in vivo-like structures that facilitate basic research surrounding several tissues and organs [2,3].

Among the 3D cell culture models, spheroids are simple aggregates of a variety of cell types, self-assembled into sphere-like formations in an environment that prevents attachment to a flat surface and promotes inter-cellular interactions [4]. Several stem-cell-derived tissue/tissue organ spheroids have been created using different spheroid systems and have been named according to the tissue/organ they are meant to resemble, including tumor spheroids, embryoid bodies, hepatospheres, neurospheres, mammospheres, and cardiospheres [5,6,7,8,9]. Nevertheless, despite the significant potential of 3D cell culture systems, many challenges remain, including the spatiotemporal distributions of oxygen, nutrients, and metabolic wastes that impact the viability of the spheroid core [10,11,12,13,14,15].

Cardiac spheroids (CSs), with greater fidelity than two-dimensional (2D) in vitro cell culture systems, exhibit biological properties and cell activities resembling those within living cardiac tissue [5,10,16]. CSs indeed provide a more accurate platform for modeling cardiac disease or drug development [17]. Embryonic stem cells (ESCs) and induced pluripotent stem cells (iPSCs) are considered the standard cell types to efficiently differentiate cardiomyocytes (CMs) in vitro, which have been widely used to generate CSs from different species, including humans [10]. When cultured in a 3D environment, rather than as 2D monolayers, pluripotent stem-cell-derived cardiomyocytes aggregate into spheroids [18] and tend to develop a more mature phenotype than in 2D conditions [19]. Although current differentiation techniques can efficiently produce beating hiPSC-derived CMs, the biomolecular, electrical, and mechanical properties of these newly differentiated cells tend to resemble those of fetal cardiomyocytes, rather than the mature cardiomyocytes of adult hearts [20]. On the other hand, adult cardiac stem/progenitor cells (CSCs) have been reproducibly used to generate floating cardiospheres that possess broad cardiac differentiation potential in vitro, including significant myogenic commitment [21,22,23,24,25,26,27,28]. Nevertheless, cardiomyocytes derived from CSCs in vitro exhibit an immature phenotype that more closely resembles embryonic cardiomyocytes rather than fully differentiated adult cardiomyocytes [21]. How and if the different spheroid formation methods, e.g., scaffold-free systems, modulate CSC-derived spheroid formation, viability, and myogenic differentiation and maturation are currently unknown.

On this premise, in this study, we generated three different CSC clones, obtained from three different mouse strains, and tested their myogenic commitment by comparing spheroid formation in 2D/3D versus 3D conditions. In particular, we compared the efficiency of scaffold-free systems, namely (i) ultra-low attachment plates and (ii) the hanging drop method, which both require a 2D/3D switch, as well as (iii) agarose micro-molds, which is an entirely 3D method, in order to produce CSs. Furthermore, these CS cell culture systems were tested for hypoxia and necrosis as a function of cell seeding density. We show that a stepwise and stage-specific myogenic differentiation assay based on a simple 2D-to-3D culture method for cardiac spheroid generation, effectively drives CSC myogenic differentiation in each tested CSC clones. The latter is improved by a low initial cell seeding density that impacts hypoxia and apoptosis within the spheroids. Moreover, performing CSC myogenic differentiation in a 2D environment after a 3D forced aggregation step, boosts cardiomyocyte differentiation. Finally, performing CS formation and ensuing myogenic differentiation exclusively in a 3D microenvironment established by agarose micro-molds, maximize cardiomyocyte yield, representing an innovative scaffold-free system that can be used to generate and simulate a more mature cardiac muscle tissue in vitro from adult stem-cell-based CSs.

## 2. Materials and Methods

### 2.1. Animals

All animal experimental procedures were approved by Magna Græcia Institutional Review Boards on Animal Use and Welfare. The mice were housed under controlled conditions of 25 °C, 50% relative humidity, and a 12 h light (6:00–18:00) and 12 h dark cycle, with water and food (containing 18.5% protein) available ad libitum. The mice were anesthetized by intraperitoneal injections of tiletamine–zolazepam (80 mg/Kg). To obtain CSC clones, hearts from C57BL/6J (stock number: 000664, Jackson Labs, Bar Harbor, ME, USA), R26mT/mG (stock number: 007576, Jackson Labs, Bar Harbor, ME, USA), and R26floxed-stop-dTomato (stock number: 007905, Jackson Labs, Bar Harbor, ME, USA) 2-month-old mice were used.

### 2.2. Myocyte-Depleted Cardiac Cell and CSC Isolation

Cardiac stem cells (CSCs) were isolated from each mouse strain, as previously described [24]. Briefly, hearts were excised, and the aorta was cannulated and hung on a retrograde perfusion system. The CSCs were isolated from the relative adult mouse heart via enzymatic dissociation using a gentleMACS Dissociator (Miltenyi Biotec, Bergisch Gladbach, Germany), and the manufacturer’s instructions were followed in order to obtain myocyte-depleted cardiac small cells [23]. To obtain Lin-/c-kit-positive CSCs, myocyte-depleted cardiac small cells were sorted with direct CD45- and CD31-negative patterns using specific anti-mouse microbeads (Miltenyi Biotec, Bergisch Gladbach, Germany). Then, FACS was used to obtain CSC-enriched CD45^neg^CD31^neg^c-kit^pos^ cells with specific fluorochrome-conjugated antibodies. All the specific antibodies are listed in Table 1.

### 2.3. Cell Culture

Freshly isolated CSCs were plated and maintained in gelatin-coated dishes in the CSC growth medium containing DMEM-F12Ham (ThermoFisher Scientific, Waltham, MA, USA) with insulin–transferrin–selenium (1%, ThermoFisher Scientific, Waltham, MA, USA), epidermal growth factor (final medium concentration: 20 ng/mL, Peprotech, London, UK), basal fibroblast growth factor (final medium concentration: 10 ng/mL, Peprotech, London, UK), leukemia inhibitory factor (final medium concentration: 10 ng/mL, Miltenyi Biotec, Bergisch Gladbach, Germany), and 1:1 ratio of Neurobasal medium (ThermoFisher Scientific, Waltham, MA, USA) containing 2mM of L-glutamine, B27 supplement (1X, ThermoFisher Scientific, Waltham, MA, USA), and N2 supplement (1X, ThermoFisher Scientific, Waltham, MA, USA) penicillin–streptomycin (1%, ThermoFisher Scientific, Waltham, MA, USA), fungizone (0.1%, ThermoFisher Scientific, Waltham, MA, USA), and gentamicin (0.1%, ThermoFisher Scientific, Waltham, MA, USA). The CSC growth medium was sterilized through a 0.22 µm pore filter in a sterile container and stored at 4 °C. The CSC growth medium was supplemented with 10% ESQ-FBS (ThermoFisher Scientific, Waltham, MA, USA). Cells were maintained at 37 °C in 21% O_2_ and 5% CO_2_. The media were replenished every 48 h and the cells were passaged at a 1:4 ratio [29].

### 2.4. Clonogenic and Spherogenesis Assay In Vitro

Single cell cloning was performed by depositing CSCs at P8 into a 96-well gelatin-coated Terasaki plate by serial dilution. Individual cells were grown in a CSC growth medium for 2 weeks until the clones were identified and expanded. A total of 10 plates were analyzed for each cell type [24,30]. For cardiac spheroid generation, the CSC clones were placed in ultra-low attachment dishes with a CSC growth medium. Cardiac spheroids were counted per plate and the number was expressed as a percentage relative to the number of plated CSCs.

### 2.5. Cardiac Spheroid Generation and Culture

#### 2.5.1. Standard Differentiation

**Figure 1:**
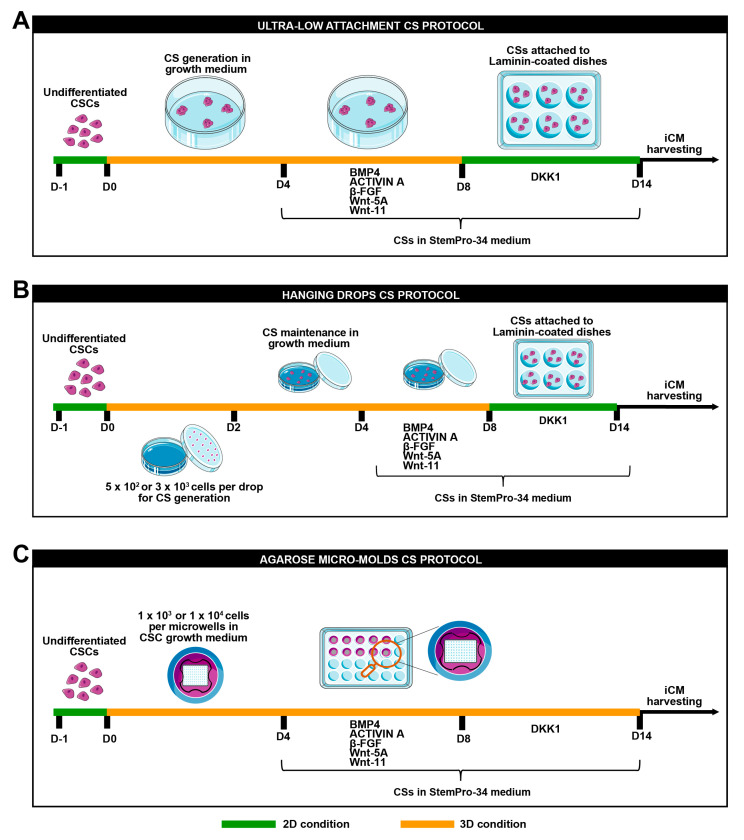
Schematic representation of cardiomyogenic differentiation protocol. (**A**) Schematic representation of ultra-low attachment cardiac spheroid protocol, (**B**) hanging drops cardiac spheroid protocol, and (**C**) agarose micro-mold cardiac spheroid protocol.

Next, 2 × 10^5^ and 2 × 10^4^ of CSC clones, respectively, were plated in 100 mm ultra-low attachment culture dishes in 6 mL of standard CSC growth medium and incubated at 37 °C under normoxia conditions (21% O_2_ and 5% CO_2_). The medium was added to the plates every two days. Cardiac spheroids started to appear after 2 days from the cell seeding and were totally formed at day 4 (D4) (Figure 1A).

#### 2.5.2. Hanging Drops

For cardiac spheroid generation, via the hanging drop method, 5 × 10^2^ or 3 × 10^3^ CSCs were placed as drops onto the inverted lid of a Petri dish. Then, 10 mL of PBS was placed in the bottom of the dish acting as a hydration chamber. Using a multichannel pipette, rows of 20 μL/drop were placed on the bottom of the dish lid in a standard CSC growth medium. Cardiac spheroids appeared after 1 day from cell seeding. On day 2, drops were collected using PBS and then transferred into 15 mL tubes. After spontaneous precipitation for 20 min at room temperature, cardiac spheroids were transferred into 100 mm ultra-low attachment culture dishes in a standard CSC growth medium and were incubated at 37 °C under normoxia conditions (21% O_2_ and 5% CO_2_) until day 4 (D4) (Figure 1B).

#### 2.5.3. Agarose Microwells Molds

A 3% agarose (ThermoFisher Scientific, Waltham, MA, USA) gel solution was prepared in PBS. The powder was dissolved in a microwave oven and the agarose microwells were fabricated in sterile conditions. In brief, the heated agarose solution was added into a MicroTissues 3D Petri dish micro-mold (in a 24-well plate format) (Merck, Darmstadt, Germany). Upon cooling at room temperature for 10 min, the agarose was removed from the molds, thus creating 24 culture inserts, each consisting of 96 microwells (diameter × height = 400 × 800 μm). The culture inserts were transferred into a 24-well plate and equilibrated in PBS overnight at 37 °C under normoxia conditions (21% O_2_ and 5% CO_2_) [31]. For cardiac spheroid generation, using the agarose mold method, 1 × 10^4^ or 1 × 10^3^ CSCs per microwell were plated. Subsequently, 1 mL of a standard CSC growth medium was added to completely cover the microwell area and the medium was incubated at 37 °C under normoxia conditions (21% O_2_ and 5% CO_2_) until day 4 (D4) (Figure 1C).

### 2.6. In Vitro Myogenic Differentiation Protocol

At day 4 (D4), cardiac spheroids were switched to a base differentiation medium: the StemPro^®^-34 SFM medium (a serum-free medium conditioned with StemPro^®^-Nutrient Supplement, ThermoFisher Scientific, Waltham, MA, USA), ascorbic acid (0.5 Mm, Sigma-Aldrich, St. Louis, MO, USA), 1-thioglycerol (4.5 × 10-4 M, Sigma-Aldrich, St. Louis, MO, USA), glutamine (2 mM), non-essential amino acids (ThermoFisher Scientific, Waltham, MA, USA), and penicillin–streptomycin (1%, ThermoFisher Scientific, Waltham, MA, USA). The base differentiation medium was implemented with BMP-4 (10 ng/mL, Peprotech, London, UK), activin-A (50 ng/mL first day and then 10ng/mL, Peprotech, London, UK), β-FGF (10 ng/mL, Peprotech, London, UK), Wnt-11 (150 ng/mL, R&D System, Minneapolis, USA), and Wnt-5a (150 ng/mL, R&D System, Minneapolis, USA). The differentiation medium was added from day 4 to day 8 (D8) when differentiating cardiac spheroids were pelleted and transferred to laminin-coated dishes (1 µg/mL), and Dkk-1 (150 ng/mL, R&D System, Minneapolis, USA) was added to the base differentiation medium from day 8 to day 14 (Figure 1) [22,30]. Differentiated cardiac spheroids were either trypsinized for RNA isolation, fixed with 4% PFA, or dissociated for FACS analysis. For monolayer single-cell myogenic differentiation, CSC clones were plated in laminin-coated dishes in a growth medium as single cells for 4 days and then they were switched in a base differentiation medium implemented with BMP4, βFGF, activin A, Wnt-11, and Wnt-5a from day 4 to day 8 and then with a base differentiation medium implemented with Dkk1 till 14 days (D14).

### 2.7. Cardiac Spheroid Size Measurements

Cardiac spheroids were photographed with a EVOS microscope (Evos XL, ThermoFisher Scientific, Waltham, MA, USA) at 10X magnification, and spheroid diameters were determined using ImageJ software 1.53t.

### 2.8. Aggregation/Disaggregation Protocol

Cardiac spheroids were collected on day 7 of the myogenic commitment and disaggregated thought enzymatic digestion with 0.05% trypsin (ThermoFisher Scientific, Waltham, MA, USA) for 5 min at 37 °C, followed by accutase incubation (ThermoFisher Scientific, Waltham, MA, USA) at 37 °C for 20 min. Half-disaggregated CSs were placed in laminin-coated dishes for single-cell monolayer culture in a differentiation medium (StemPro^®^-Nutrient Supplement with BMP-4, activin-A, β-FGF, Wnt-11, and Wnt-5a) for 24 h and then till day 14 with a base differentiation medium implemented with DKK1. The other half-disaggregated CSs were replaced in suspension for 24 h in a differentiation medium (StemPro^®^-Nutrient Supplement with BMP-4, activin-A, β-FGF, Wnt-11, and Wnt-5a) and then attached in laminin-coated dishes and switched to a base differentiation medium implemented with DKK1 till D14 when the cells were collected and processed for RNA extraction.

### 2.9. Flow Cytometry

To assess CSC phenotypic identity and cardiac-related gene expression, FACSCanto II (BD, New Jersey, USA) and Fortessa X20 (BD, New Jersey, USA) were used [22,24]. FACS data were analyzed using FlowJo software V.X TREE STAR. Specific antibodies used are shown in Table 1. Appropriate labeled isotype controls were used to define the specific gates.

### 2.10. Quantitative RT-PCR (qRT-PCR)

RNA was extracted from the collected samples using TRIzol reagent (ThermoFisher Scientific, Waltham, MA, USA) and quantified using a Nanodrop 2000 spectrophotometer (ThermoFisher Scientific, Waltham, MA, USA). Reverse transcription was performed with 0.5–1 µg of RNA using a high-capacity cDNA kit (ThermoFisher Scientific, Waltham, MA, USA). Quantitative RT-PCR was performed using TaqMan primer (ThermoFisher Scientific, Waltham, MA, USA) (Table 2) using a CFX Opus RealTime PCR System (Biorad, CA, USA). The data were processed with the ΔCt method using CFX Manager Software 2.3 (Biorad, California, USA) and mRNA was normalized to the housekeeping gene, known as GAPDH. All reactions were carried out in technical triplicate.

### 2.11. Cardiac Spheroid Harvesting, Embedding, and Sectioning

Cardiac spheroids were collected at each time point of the differentiation protocol, and were fixed using 4% paraformaldehyde (PFA, #P6148, Sigma-Aldrich, St. Louis, MO, USA) for 30 min at room temperature. Cardiac spheroids were firstly embedded in a 1% (*w*/*V*) agarose solution (ThermoFisher Scientific, Waltham, MA, USA). The resulting agarose block was incubated in sucrose 30% (*w*/*V*) overnight and embedded in an optimal cutting temperature compound (O.C.T). The samples were cut in 5 µm cross-sections and analyzed until the centermost of cardiac spheroid.

### 2.12. Immunofluorescent Staining

For immunofluorescent staining, the slides were washed with PBS and then incubated for 1 h with a primary antibody against cTnI (1:100 dilution, Abcam, Cambridge, UK), c-kit (1:50 dilution; R&D Systems, Minneapolis, USA), HIF-1α (1:100 dilution; Abcam, Cambridge, UK), Ki67 (1:50 dilution; Dako, Santa Clara, CA, USA) connexin43 (1:100 dilution; Cell Signaling, Massachusetts, USA), and TUNEL (In Situ Cell Death Detection Kit, Fluorescein, Sigma-Aldrich, St. Louis, MO, USA). The slides were then washed and incubated with respective anti-mouse IgG, anti-rabbit IgG, or anti-goat IgG secondary antibodies (1:100 dilution; Jackson Immunoresearch, Ely, Cambridgeshire, UK) for 1 h at 37 °C. The nuclei were counterstained with the DNA binding dye, known as 4, 6-diamidino-2-phenylindole (DAPI, Sigma-Aldrich, St. Louis, MO, USA), at 1 µg/mL. The slides were mounted in a VECTASHIELD antifade mounting medium and cardiac spheroids sections were analyzed using confocal microscopy (TCS SP8, Leica Microsystems, Wetzlar, Germany) [32,33,34]. To evaluate cellular apoptosis and hypoxia, the centermost CS sections were obtained by cutting at least 20 sections of 5 µm each for CS with a diameter of 200 µm (the number of cut sections to reach the centermost was dependent on the CS diameter). The specific antibodies used are shown in Table 1.

### 2.13. Masson’s Trichrome Staining

Cardiac spheroid sections were stained with Masson’s Trichome (PolySciences, Inc., Warrington, PA, USA) for the quantification of muscle cells and fibers. Specifically, the slides containing sections were fixed in Bouin’s fixative solution at 60 °C for 1 h and then treated with Weigert’s iron hematoxylin working solution for 10 min, with Biebrich scarlet–acid fuchsin for 5 min, before being transferred to phosphotungstic/phosphomolybdic acid for 5 min, aniline blue solution for 5 min, and then to 1% acetic acid for 1 min. Then, the sections were dehydrated in 95% alcohol for 2 min, cleared with 2 changes of xylene, and mounted. Sections were imaged using light microscopy (Evos XL, ThermoFisher Scientific, Waltham, MA, USA).

### 2.14. Statistical Analysis

Statistical analysis was performed with GraphPad Prism version 6.00 for Macintosh (GraphPad Software 6). The quantitative data are reported as mean ± SD and binary data based on counts. The significance between the 2 groups was determined using Student’s t test or paired *t* test as appropriate. To draw comparisons between multiple groups, the ANOVA was used. A *p* value of <0.05 was considered significant. Bonferroni’s post-hoc method was used to locate the differences. In these cases, the type 1 error (α = 0.05) was corrected by the number of statistical comparisons performed. For the in vitro cell and molecular biology experiments with an *n* = 4 sample size, giving the low number of the sample, the Kruskal–Wallis test (for comparisons between multiple groups) and the Mann–Whitney U test (for comparison between 2 groups) were performed.

## 3. Results

### 3.1. Isolation of True Multipotent Adult Cardiac Stem Cells

To assess the phenotypic and transcriptional changes occurring during CSC myogenic specification, cardiomyocyte-depleted cardiac cell preparations were obtained from wild-type C57BL/6J mice, as well as from the Cre reporter ROSA26^mT/mG^ and ROSA26^floxed-stop/dTomato^ mice (both on a C57BL/6J background), through mechanical (gentleMAC Dissociator) dissociation in collagenase type-II-based digestion buffer, followed by gravity separation [23,24]. These three mouse strains were chosen to eventually obtain CSCs and CSC-derived CSs useful for cell tracking in vivo upon transplantation [23,35,36]. Cardiomyocyte-depleted cardiac cells were efficiently depleted of CD45- and CD31-expressing cells by magnetic-activated cell sorting (MACS) [23,24] and were then FACS-sorted to obtain a pure (over 95%) population of CD45^neg^/CD31^neg^/c-kit^pos^ cardiac cells [23,24], which we shown to be enriched for multipotent CSCs [23,24]. These cells were then grown in vitro for eight passages [24] and deposited at a single-cell level in 96 wells to obtain respective single-cell-derived clones. Fourteen days later, a single CSC clone from each mouse strain (hereafter, WT-, mT/mG-, and Tomato-CSCs) was randomly picked and used in this study [24]. These three CSC clones had a phenotypic and transcriptomic identity that is similar to, if not indistinguishable with CSC clones that we previously characterized [23,24] (Supplementary Table S1).

The three CSC clones proved to have a similar amplification capacity, sub-clonogenic ability, and primary and secondary spherogenic potential, which made them a reliable source of multipotent CSCs to be tested for their CS formation and cardiomyocyte differentiation capacity in vitro.

### 3.2. The 2D-to-3D Culture Switch Modulates CSC Plasticity and Cardiomyocyte Differentiation Potential In Vitro

To compare the myogenic commitment and specification of the three CSC clones in three different scaffold-free methods, namely ultra-low attachment surface, hanging drops, and agarose micro-mold wells (Figure 1A), we first characterized the phenotypic and transcriptional changes occurring during the switch from 2D to 3D culture conditions, and then during CSC differentiation on the extracellular matrix (ECM)-derived laminin coating using the cardiosphere differentiation assay that we previously established and verified in our lab [21,23,24,37], in line with previous protocols established on iPSCs [18,19,20,38].

**Figure 2:**
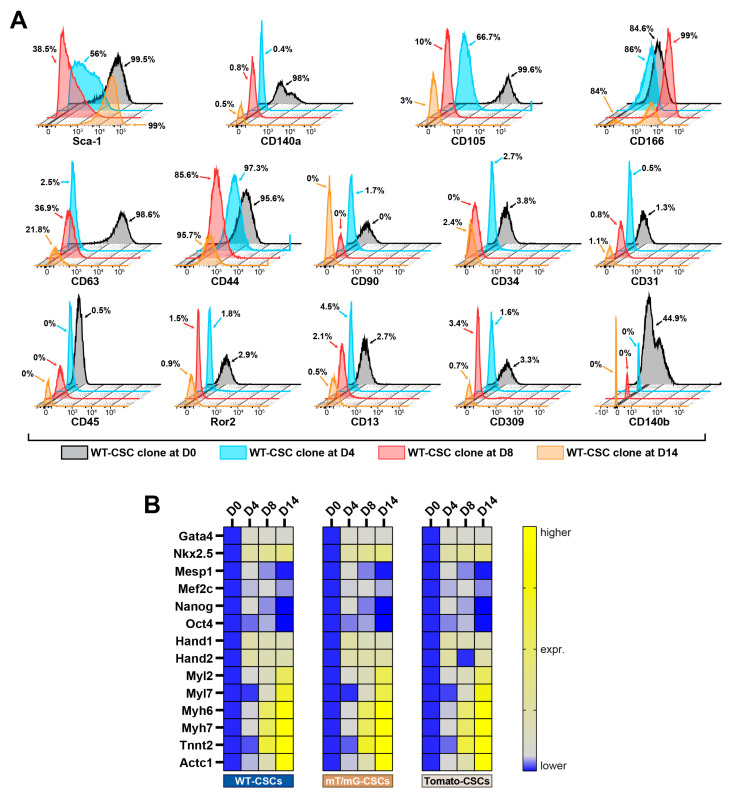
Time course of the phenotypic and transcriptomic characterization of CSC-derived cardiac spheroids. (**A**) FACS fluorescence histograms describing cell surface markers expression and modulation at D0, D4, D8, and D14 of the myogenic commitment in the WT-CSC clone (representative of *n* = 3). (**B**) Heat map showing qRT-PCR analysis of genes involved in biological processes as pluripotency, cardiac development, and muscle contraction in differentiating WT-CSCs, mT/mG-CSCs, and Tomato-CSCs at D0, D4, D8, and D14 of the myogenic commitment protocol. The data are representative of fold changes over the respective baseline value (D0) of each mRNA. The color scale indicates changes in Ct (threshold cycle) relative to the normalized GAPDH control (representative of *n* = 3). The data are expressed as mean ± S.D.

Two-dimensional cell monolayers of proliferating CSC clones were enzymatically detached from gelatin-coated dishes (day 0, D0) and then plated in CSC growth media at a density of 2 × 10^5^ cells/6 mL in 100 mm^2^ ultra-low attachment plates for 4 days to facilitate their self-aggregation in 3D floating spheres (Figure 1A). On day 4 (4D), self-aggregated CSC-derived CSs were isolated to be stored for further analysis or replated in ultra-low attachment dishes from day 4 to day 8 in a base differentiation medium (StemPro34) conditioned by the cardiac morphogens BMP-4 (10 ng/mL), activin A (50 ng/mL), basic fibroblast growth factor (β-FGF) (10 ng/mL), WNT-11 (150 ng/mL), and WNT-5a (150 ng/mL) (Figure 1A). This second step allows the modulation of activin/nodal and BMP signaling at the proper time, as typically occurs during heart development in vivo [21,22,23]. The CS samples were then collected at day 8 (D8) or further processed in the third and final differentiation step, when differentiating CSs were transferred to laminin-coated dishes in the StemPro34 medium with Dickkopf-related protein (DKK-1) (150 ng/mL) added every two days from day 8 till day 14 to persistently inhibit the canonical Wnt/β-catenin pathway [21,22,23,24,29] (Figure 1A). During this step, on day 10, when CSC-derived CSs adhere to a laminin coating, they start spontaneous contractions with autonomous beating, as previously reported [21,23,24]. Contracting CSC-derived CSs were then collected at day 14 (D14), and named as CSC-induced CMs (iCMs). Cell samples obtained at different time points were processed for RNA extraction or fixed in 4% paraformaldehyde for immunohistochemistry analysis. Alternatively, CSs were disaggregated through enzymatic dissociation to obtain a single-cell suspension for flow cytometry analysis in order to assess the expression of surface markers and for gene expression, as reported in Figure 2.

Flow cytometry analysis shows that the switch from the 2D monolayer to the 3D spheroid culture (D0 vs. D4 through D8 timepoints) modified the expression of several CSC membrane markers (Figure 2A and Supplementary Table S1). At D0, CSC clones were uniformly positive for Sca-1 (Figure 2A and Supplementary Table S1), but at D4 spheroid formation by cell self-aggregation significantly decreased Sca-1 expression, which remained downregulated during myogenic commitment in suspension (D8) (Figure 2A and Supplementary Table S1). Sca-1 was then totally re-expressed in CSs attached to laminin coating dishes at D14 of the myogenic commitment (Figure 2A and Supplementary Table S1). CD105 expression decreased over time in the culture, while CD166 expression remained high during all the steps of CSC differentiation (Figure 2A and Supplementary Table S1). On the other hand, CD63 expression decreased at D4 and then increased again through the following steps of the differentiation protocol (D14, Figure 2A and Supplementary Table S1). CSC clones in the 2D monolayer culture at D0 were homogenously positive for CD140α and β (Figure 2A and Supplementary Table S1); their expression was significantly reduced by the 2D-to-3D culture switch for CS formation (D4) and remained practically absent at D14 (Figure 2A and Supplementary Table S1). CD45, CD31, CD34, and CD309 were nearly absent during the different steps of CS formation and differentiation (Figure 2A and Supplementary Table S1).

Remarkably, the mRNA levels of pluripotency genes and the main cardiac transcription factors Gata-4, Nkx2.5, Mesp1, Mef2c, Nanog, Oct4, Hand1, and Hand2 were upregulated at D4 as a result of the 2D-to-3D culture switch for CS formation (Figure 2B and Supplementary Figure S1A). These data indicate that the switch from 2D-to-3D culture with CSC self-aggregation provides cues that confer enhanced plasticity and cardiac tissue potential to CSC-derived CS in vitro.

mRNA levels of cardiac contractile proteins, such as Tnnt2, Actc1, Mhy7, and Mhy6, started to be faintly detectable in CSs from D4 (Figure 2B and Supplementary Figure S1A). These genes reached the highest levels of expression in iCMs at D14 (Figure 2B and Supplementary Figure S1A).

Thus, the transcriptomic profile of CSC-derived CSs and their myogenic commitment in iCMs, closely resembled the activation of transcriptional program from mesodermal specification to heart development [21]. Of interest, the modulation of Sca-1 expression in CSC-derived CS formation and differentiation, followed a pattern similar to its in vivo expression during cardiac development, when Sca-1 is absent, and during adult heart maturation, when Sca-1 is significantly expressed [39,40,41].

### 3.3. Three-Dimensional CSC Self-Aggregation Is Necessary to Guide Robust CS Myogenic Commitment

**Figure 3:**
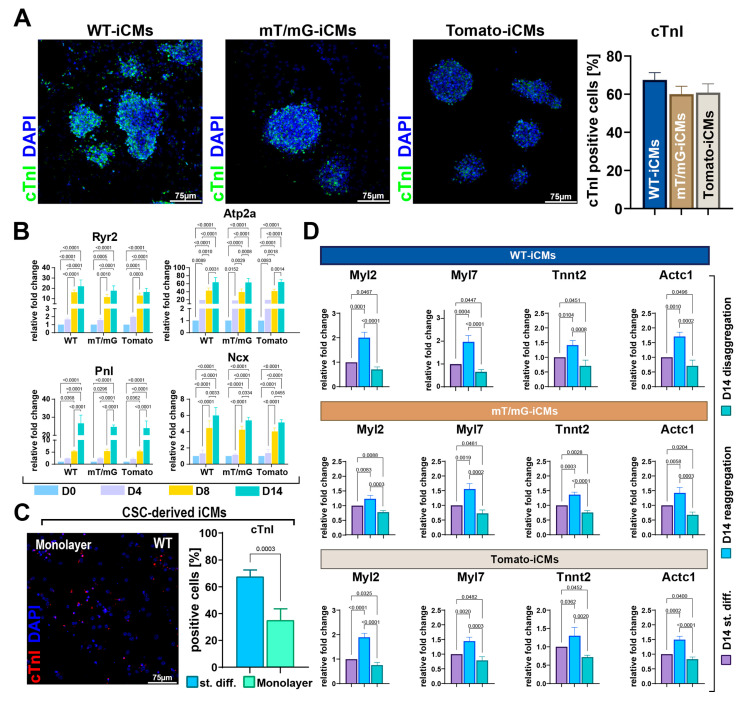
Myogenic specification of CSs as compared to CSC monolayers and as a function of self-aggregation. (**A**) Representative confocal microscopy images and bar graph showing cTnI-positive cells in WT-iCMs, mT/mG-iCMs and Tomato-iCMs at D14 of cardiomyogenic differentiation (cTnI, green, DAPI/blue. Scale bar = 75 μm) (representative of *n* = 6). (**B**) Bar graphs showing the cumulative qRT-PCR data of genes involved in calcium handling in WT-CSCs, mT/mG-CSCs and Tomato-CSCs at D0, D4, D8, and D14 of cardiomyogenic differentiation (*n* = 3 for each clone). (**C**) Representative confocal microscopy image of WT-iCMs obtained in monolayer culture conditions (cTnI, red; nuclei/DAPI/blue. Scale bar = 75 μm). The bar graph shows the percentage of cTnI-positive iCMs at D14 obtained from the three CSC-clone-derived CSs using the ultra-low attachment protocol (named as standard differentiation, st.diff.) compared to the differentiation of 3 clones in monolayer culture conditions (*n* = 3 for each clone). (**D**) Bar graphs showing cumulative qRT-PCR of cardiac-related gene expression in WT-iCMs, mT/mG-iCMs, and Tomato-iCMs at D14 of the myogenic commitment after CS disaggregation and reaggregation at D8 compared to iCMs obtained after ultra-low attachment CS protocol (i.e., standard differentiation) (*n* = 3 for each clone). The data are expressed as mean ± S.D.

Differentiating CSC clones upregulated cardiac troponin expression during CS formation and differentiation, and the percentage of troponin-positive cells during the myogenic commitment of CSs reached roughly 65% at D14 of the differentiation process (Figure 3A). Moreover, from D8 to D14, we observed a ~two-fold higher expression in genes involved in calcium handling as the ryanodine receptor (Ryr2), SERCA2a (Atp2a), and the Na(^+^)/Ca(^2+^) exchanger (Ncx), and a ~six-fold higher expression in the phospholamban (Pnl) gene, indicating a progressive maturation of CSC-derived CSs during myogenic commitment (Figure 3B). On this basis, we assessed the importance of 3D CS formation in the myogenic differentiation of CSCs.

First, we performed CSC myogenic differentiation as a single-cell monolayer culture, simply replacing growth medium with the differentiation medium, as shown in Figure 1A. Then, we directly plated the CSCs at D0 on laminin-coated dishes without switching them from a 2D condition to an ultra-low attachment 3D culture condition. When CSCs were differentiated in this entirely 2D culture condition, at D14, we detected 35 ± 8% of troponin-positive cells, demonstrating a reduced CSC myogenic commitment as compared to the switch from 2D to 3D culture conditions (Figure 3C).

Then, to investigate whether CSC self-aggregation promotes cardiomyocyte differentiation and whether CS disaggregation reduces it, CSs were collected on day 7 of the CS assay protocol (Figure 1A) and disaggregated through trypsin/accutase treatment. Half of the disaggregated CSs were then directly placed in adhesion in laminin-coated dishes as a single-cell monolayer culture and left for 24 h in differentiation media (StemPro34 medium implemented with BMP-4, activin A, β-FGF, WNT-11, and WNT-5a); the remaining disaggregated CSs were first left to re-aggregate in suspension for 24 h in the same media as above, and then both the disaggregated and reaggregated CSs were plated in laminin-coated dishes in the differentiation medium conditioned with DKK1 (Figure 1A). At D14, disaggregated and re-aggregated CSCs were collected and processed for RNA extraction and compared for contractile gene expression with iCMs from the standard CS differentiation protocol. At D14, RT-PCR analysis on reaggregated CSs revealed that the expression level of Myl2, Myl7, Actc1, and Tnnt2 was approximately two-fold higher than that detected in disaggregated cells (Figure 3D). Importantly, the expression level of these mRNAs in disaggregated cells at D14 was lower than that detected at D14 during the standard differentiation protocol (Figure 3D). Conversely, Myl2, Myl7, Actc1, and Tnnt2 mRNA levels were higher in reaggregated cells at D14 than those detected at D14 during the standard differentiation protocol (Figure 3D).

Thus, CSC 3D self-aggregation in CSs promoted a robust CSC myogenic differentiation into cardiomyocytes. Moreover, CS reaggregation further fostered CSC myogenic specification and could represent an effective strategy to promote iCM maturation in vitro.

### 3.4. CS Size Is Crucial for Its Robust Myogenic Commitment and Cardiomyocyte Differentiation

As demonstrated in human embryonic stem cell (hESC)-derived spheroids, the efficiency of cell differentiation into cardiac lineage is dependent on spheroid size [42]. In general, CS size can be controlled by initial cell seeding density. Thus, in our system, we assessed the importance of cell number in CS formation and size by altering the initial seeding density of CSCs in the switch from the 2D culture condition (D0) to the 3D culture condition (D4 and D8).

To assess whether cell seeding density affects myogenic differentiation and then cardiac-related gene expression, we reduced the cell number used for CS formation by one order of magnitude by placing 2 × 10^4^ cells in same condition shown above with 2 × 10^5^ cells.

**Figure 4:**
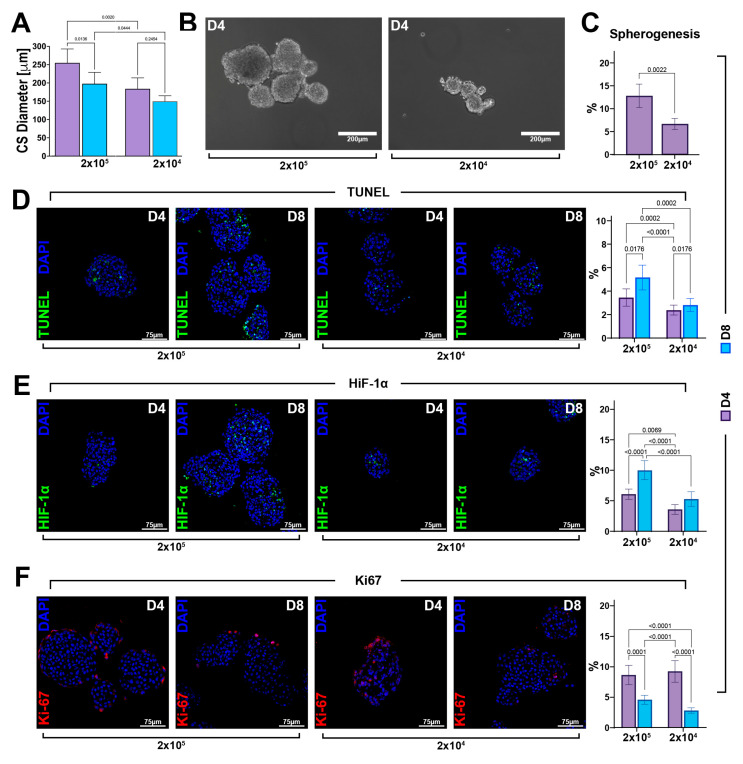
The cell viability of CSC-derived CSs depends on the cell seeding number and ensuing spheroid size. (**A**) Bar graph showing cardiac spheroid diameters at D4 and D8 generated by planting 2 × 10^5^ and 2 × 10^4^ cells per plate (representative of *n* = 3). (**B**) Representative light microscopy images of cardiac spheroids obtained by planting, respectively, 2 × 10^5^ and 2 × 10^4^ cells per plate. (scale bar = 200 μm) (representative of *n* = 6). (**C**) Bar graph showing the percentage of CSs generated of the WT-CSC clone by planting 2 × 10^5^ and 2 × 10^4^ cells per plate (representative of *n* = 3). (**D**–**F**) Representative confocal microscopy images and bar graph showing the percentage of apoptotic TUNEL^pos^ cells, hypoxic HIF-1α^pos^ cells, and proliferating Ki67^pos^ cells in the CS core at D4 and D8 of the myogenic commitment in 2 × 10^5^ and 2 × 10^4^ seeded cells. (TUNEL, green; HIF-1α, green; Ki67, red; nuclei/DAPI/blue; scale bar = 75 μm) (representative of *n* = 6). The data are expressed as mean ± S.D.

Morphologically, cardiac spheroids derived from 2 × 10^5^ and 2 × 10^4^ cells appeared equally spherical at D4. The CS diameter revealed that cell seeding affects cellular aggregation. Indeed, we detected a significative difference in CS size, with a diameter reduction of 61 ± 10 µm at D4 in the group with a lower cell seeding (Figure 4A,B). Similarly, the percentages of formed CSs at D4 were ~13 and 7%, respectively, for 2 × 10^5^ and 2 × 10^4^ seeded cells (Figure 4C). Importantly, from D4 to D8, we observed a decrease in CS size in both 2 × 10^5^ and 2 × 10^4^ seeded cell groups, which accounted for a reduction of 57 µm in the diameter of the CS derived from 2 × 10^5^ seeded cells when compared to a 34 µm reduction in the diameter of the CS derived from 2 × 10^4^ seeded cells, suggesting that time duration in culture affects CS size depending on the initial cell seeding number (Figure 4A).

A high number of apoptotic cells has been previously reported within the “hypoxic core” of spheroids due to a lower distribution of oxygen and nutrient [11,12,13,14,15,27]. Based on this observation, we hypothesized that the reduction in CS size was linked to cell death events. To this aim, fixed and OCT-embedded CSs were cut in a sequential section to analyze the CS centermost. Once in the spheroid core, we assessed the number of TUNEL-positive cells in CSs from D4 to D8 generated in higher and lower cell seeding conditions. We found an increasing number of apoptotic cells in CSs generated using 2 × 10^5^ cells, reaching 5.1 ± 1% at D8 of the cardiac differentiation protocol when compared to 2.8 ± 0.5% of apoptotic-positive cells counted in the CS generated from 2 × 10^4^ cells per plate (Figure 4D). These data indicated that cell seeding density affects cell viability within the spheroids and that cell death was dependent on the number of cells used to form CSs.

We then measured the hypoxic microenvironment within the spheroid core assessing the expression of the hypoxia inducing factor, HIF-1α. The number of HIF-1α-positive cells progressively increased in CSs generated using both 2 × 10^4^ and 2 × 10^5^ CSCs. However, at D8, there were ~10% HIF-1α-positive cells in the higher cell seeding condition, while at same time point in 2 × 10^4^ cells per plate, we observed a lower percentage (~5%) of HIF-1α-positive cells (Figure 4E). On the other hand, on the outskirt of the cardiac spheroid core, cells were similarly proliferative in each cell seeding condition at D4, as well as D8 of the CS assay protocol, as identified by the Ki67 expression (Figure 4F).

**Figure 5:**
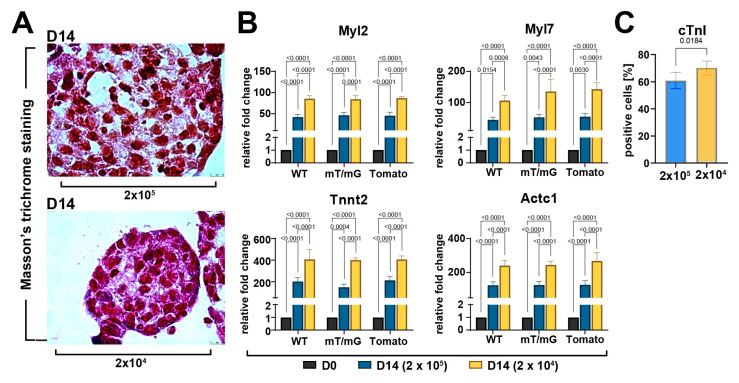
Myogenic commitment of CSC-derived CSs depends on the cell seeding number and ensuing spheroid size. (**A**) Representative light microscopy images of Masson’s trichrome-stained CSs at D14 display an increased amount of muscle cells and fibers at D14 in 2 × 10^4^ cells seeded cells compared to 2 × 10^5^ (scale bar = 10μm) (representative of *n* = 3). (**B**) Bar graphs showing cumulative qRT-PCR data of the expression levels of cardiac-related genes in WT-, mT/mG-, and Tomato-iCMs at D14 of the myogenic commitment derived, respectively, from 2 × 10^5^ and 2 × 10^4^ seeded cells (*n* = 3 for each clone). The data are representative of fold changes over the baseline value (D0) of each mRNA, respectively. (**C**) Bar graphs showing cumulative data of the number of cardiac troponin-positive cells at D14 of the myogenic commitment in iCMs from the 3 CSC-clone-derived CSs derived, respectively, from 2 × 10^5^ and 2 × 10^4^ seeded cells (*n* = 3 for each condition). The data are expressed as mean ± S.D.

Based on Masson’s trichrome staining, we then evaluated whether cell seeding number modulated muscle cell density and fiber formation in CSs. An increased amount of muscle cells and fibers at D14 was observed in the 2 × 10^4^ seeded cells as compared to the 2 × 10^5^ seeded cells group (Figure 5A). Moreover, we analyzed the mRNA expression level of cardiac-related genes in higher and lower cell seeding conditions at D14 as compared to D0. We detected ~two-fold higher expression levels of Tnnt2, Actc1, Myl7, and Myl2 in cardiac spheroids derived from 2 × 10^4^ seeded cells collected at D14 of the differentiation protocol compared to cardiac spheroids from 2 × 10^5^ seeded cells (Figure 5B). Accordingly, differentiating CSs derived from 2 × 10^4^ seeded cells showed a higher percentage of troponin-positive cells at D14 of the differentiation process when compared to CSs from 2×10^5^ seeded cells (Figure 5C).

Overall, these data show that the initial cell number affect the spheroid size and cell viability which are fundamental for the balanced growth and robust myogenic differentiation of CSCs in vitro.

### 3.5. Forced Aggregation by Hanging Drop Fosters CSC-Derived CS Cardiomyocyte Differentiation

We then assessed the importance of the forced cell aggregation and cell seeding number for CSC-derived CS formation and ensuing myogenic differentiation using the hanging drops method [6], a straightforward approach where cells are suspended in droplets of the medium and cell assembly into 3D aggregates is assisted and partly forced by gravity (Figure 1B).

**Figure 6:**
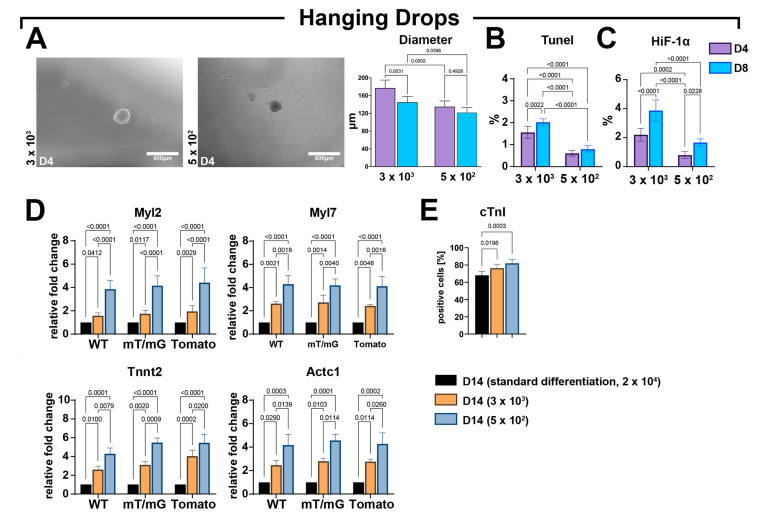
Forced aggregation into hanging drops fosters an improved CSC-derived CS myogenic differentiation. (**A**) Representative light microscopy images of cardiac spheroids at D4 and bar graph showing CS diameters at D4 and D8 of the myogenic commitment using 3 × 10^3^ or 5 × 10^2^ seeded cells per drop (representative of *n* = 3) (scale bar = 400 μm). (**B**,**C**) Bar graphs showing the percentage of TUNEL^pos^ and HIF-1α^pos^ cells using, respectively, 3 × 10^3^ and 5 × 10^2^ seeded cell per drop at D4 and D8 (representative of *n* = 3). (**D**) Bar graphs showing the cumulative qRT-PCR data of the expression levels of cardiac-related genes in WT-, mT/mG-, and Tomato-iCMs at D14 using, respectively, 3 × 10^3^ and 5 × 10^2^ seeded cells per drop as compared to the standard differentiation protocol (ultra-low attachment 2D/3D switch using 2 × 10^4^ seeding cells) (*n* = 3 for each clone). The data are representative of fold changes over the respective baseline value (D14 standard differentiation at 2 × 10^4^ seeded cells) of each mRNA. (*n* = 3 for each clone). (**E**) Bar graphs showing cumulative data of the number of cardiac troponin-positive cells at D14 of the myogenic commitment in iCMs from the 3 CSC-clone-derived CSs using, respectively, 3 × 10^3^ and 5 × 10^2^ seeded cell per drop as compared to standard differentiation protocol (ultra-low attachment 2D/3D switch using 2 × 10^4^ seeding cells) (*n* = 3 for each condition). The data are expressed as mean ± S.D.

We generated hanging drops containing 5 × 10^2^ or 3 × 10^3^ cells per drop. After 48 h, the CSs were already generated, and therefore transferred for two more days in ultra-low attachment plates in a CSC growth medium. The latter medium was replaced by the differentiation medium for the next 4 days and then hanging-drop-derived CSs were plated in laminin dishes up to 14 days (Figure 1B). After 4 days in the growth medium, the size of CSs generated using 3 × 10^3^ cells was 177 ± 18 μm, reaching 145 ± 13 μm at D8 (Figure 6A). In CSs generated using 5 × 10^2^ cells, the diameter at D4 was 135 ± 13 μm, reaching 122 ± 11 μm at D8 (Figure 6A). Importantly, hanging drops filled with 5 × 10^2^ cells displayed a reduced number of TUNEL-positive as well as HIF-1α-positive cells compared with hanging drops containing 3 × 10^3^ cells (Figure 6B,C).

CSs obtained from hanging drops at the end of the myogenic protocol were compared with differentiated CSs derived from ultra-low attachment plates following the standard protocol of laminin-coated dishes (2D/3D switch) using 2 × 10^4^ cells (Figure 6D). The higher expression levels of Myl2 Myl7, Tnnt2, and Actc1 were detected in CSs derived from the hanging drop protocol when compared to CSs from standard protocol (Figure 6D). Furthermore, at D14, the expression levels of the four myogenic genes in differentiated CSs from the hanging drop protocol were higher in the lower cell seeding condition when compared to the higher cell seeding condition (Figure 6D). Accordingly, the hanging drop protocol increased the number of cardiac troponin-positive cells in differentiated CSs at D14 when compared with CSs differentiated with the standard protocol (Figure 6E). The number of troponin-positive cells within CSs formed with the hanging drop force aggregation was higher in the lower cell seeding condition when compared to the higher cell seeding condition (Figure 6E).

Overall, these data indicate that cell seeding number, together with the forced cell assembly to produce CSs, impacts its cardiomyocyte differentiation in vitro.

### 3.6. CSC-Derived CS Growth and Differentiation Solely in 3D Culture Condition Maximizes Cardiomyocyte Yield

**Figure 7:**
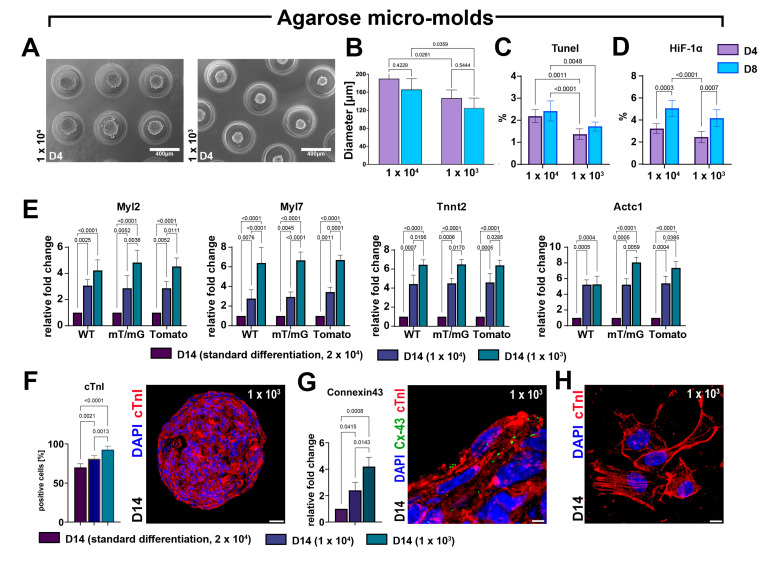
The CS formation under exclusive 3D conditions using agarose micro-molds maximizes myogenic differentiation. (**A**,**B**) Representative light microscopy images of cardiac spheroids at D4 and bar graph showing CS diameters at D4 and D8 of myogenic commitment when generating with 1 × 10^4^ or 1 × 10^3^ seeded cells per agarose micro-molds (representative of *n* = 3). (**C**,**D**) Bar graphs showing the percentage of TUNEL^pos^ and HIF-1α^pos^ cells using, respectively, 1 × 10^4^ or 1 × 10^3^ cells per agarose micro-molds (representative of *n* = 3). (**E**) Bar graphs showing the cumulative qRT-PCR data of the expression levels of cardiac-related genes in WT-, mT/mG-, and Tomato-iCMs at D14 using, respectively, 1 × 10^4^ or 1 × 10^3^ cells per agarose micro-molds as compared to the standard differentiation protocol (ultra-low attachment 2D/3D switch using 2 × 10^4^ seeding cells). The data are representative of fold changes over the respective baseline value (D14 standard differentiation at 2 × 10^4^ seeded cells) of each mRNA. (*n* = 3 for each clone). (**F**) Bar graphs showing cumulative data of the number of cardiac troponin-positive cells at D14 of the myogenic commitment in iCMs derived from the 3 CSC-clone-derived CSs using, respectively, 1 × 10^4^ or 1 × 10^3^ cells per agarose micro-molds as compared to the standard differentiation protocol (ultra-low attachment 2D/3D switch using 2 × 10^4^ seeding cells) (*n* = 3 for each condition) (scale bar = 25 μm). (**G**) Bar graph and representative confocal image showing, respectively, the qRT-PCR data and the immunocytochemistry of the expression levels of connexin-43 (Cx-43) gene and protein in iCMs derived from 3 CSC-clone-derived CSs at D14 of the differentiation process using, respectively, 1 × 10^4^ or 1 × 10^3^ cells per agarose micro-molds as compared to the standard differentiation protocol (ultra-low attachment 2D/3D switch using 2 × 10^4^ seeding cells) (scale bar = 5 μm). (**H**) Representative confocal image showing definitive sarcomeric organization in iCMs derived from CSC-derived CSs at D14 of the differentiation process using 1 × 10^3^ cells per agarose micro-molds (scale bar = 5 μm). The data are expressed as mean ± S.D.

To further assess the significance of 3D culture systems in CSC differentiation towards the myogenic commitment, we applied an in vitro method designed for fast and uniformly sized CS formation consisting of agarose micro-molds [31], containing 96 microwells (diameter x height = 400 × 800 μm), in which we placed 1 × 10^4^ or 1 × 10^3^ CSCs per well (Figure 1C). Using this method, we were able to assess CSC-derived CS formation and its myogenic differentiation entirely in a 3D culture condition, manipulating only medium changes without perturbing the 3D state. CS size was significantly reduced in agarose micro-molds wells containing 1 × 10^3^ cells compared to wells plated with 1 × 10^4^ cells (Figure 7A,B) at both D4 and D8. However, the sizes within each group did not show significant changes from D4 to D8 during culture (Figure 7A,B). The number of TUNEL-positive as well as HIF-1α-positive cells in agarose micro-molds was dependent on initial cell seeding (Figure 7C,D).

More importantly, at D14 in the agarose micro-molds wells, we detected a significant and cell-seeding-dependent increase in the expression level of Tnnt2, Actc1, Myl7, and Myl2 compared with standard 2D-to-3D myogenic differentiation protocol performed using 2 × 10^4^ cells per plate (Figure 7E). Accordingly, the agarose molds protocol increased the number of cardiac troponin-positive cells in differentiated CSs at D14 when compared with CSs differentiated with the standard protocol (Figure 7F). The number of troponin-positive cells within CSs formed using the agarose micro-mold deposition method was higher in the lower cell seeding condition compared to the higher cell seeding condition (Figure 7F). The fold increase in cardiac gene expression and the higher number of troponin-positive iCMs in the agarose micro-molds compared to standard differentiation protocol (see Figure 6B and Figure 5B) reveals that the CS myogenic differentiation protocol was successfully conducted entirely in 3D conditions, also improving the hanging-drop-based protocol in terms of enhancing cardiomyocyte commitment. This progressive maturation of CS-derived iCMs differentiated in an entirely 3D environment was further shown by the significant increase in mRNA and the protein expression of the gap junction Connexin-43, which was higher at D14 in agarose micro-molds seeded with 1 × 10^3^ CSCs per well (Figure 7G). Concurrently, iCMs derived in the latter conditions, when placed in laminin-coated dishes for 24 h, show defined and well-structured sarcomeres (Figure 7H).

These findings suggest that CSC self-aggregation in a purely 3D condition promotes more effective CS formation and growth, thereby maximizing their cardiomyocyte differentiation potential in vitro.

## 4. Discussion

The main findings emanating from the present study are as follows: (i) the 2D-to-3D culture switch per se modulates CSC plasticity and myogenic commitment; (ii) CSC 3D self-aggregation is necessary for robust CS myogenic differentiation into iCMs; (iii) CS reaggregation further fosters CSC myogenic specification, representing an effective strategy to promote iCM maturation in vitro; (iv) cell seeding and ensuing CS size are fundamental for the balanced growth and robust myogenic differentiation of CSs; (v) CSC-derived CSs obtained exclusively in a 3D culture environment maximizes the cardiomyocyte yield.

In regenerative medicine, tissue-specific adult CSCs are a potential cell source to replenish cardiomyocytes (CMs) lost in response to cardiac disease [43,44,45,46]. Their reparative and regenerative properties depend on their CM differentiation potential. The latter has been the topic of several efforts made to increase the efficiency of CM generation and maturation from these cells in vitro and in vivo [45,47,48,49]. In this study, we described a protocol consisting of the stepwise addition of growth factors and/or small molecules, collectively known as cardiac morphogens, that reproducibly and efficiently induce CSC cardiomyocyte differentiation in 3D cardiac spheroids. Spheroid culture is a versatile and powerful tool used in many areas of regenerative medicine that, in concert with established techniques, could provide new insights and solutions for several chronic degenerative diseases [4,16,50,51]. Spheroids are complex in vitro structures that better mimic the in vivo cell environment within mammalian tissue when compared to conventional two-dimensional culture systems in vitro [52]. Spheroids are the basis for structured organoid generation, which are progressively filling the gap towards the achievement of reliable miniaturized organ models “in a dish” [5].

In this study, we demonstrated that when differentiated as monolayers cell cultures, multipotent adult CSCs are barely efficient in cardiomyocyte differentiation when primed with the same cardiac morphogens used for cardiac spheroid generation, showing that cell-to-cell interaction in 3D culture are key for efficient CM differentiation in vitro. Accordingly, we describe a CSC-derived CS generation protocol and its myogenic differentiation consisting of three main steps: (i) the generation of CSs from undifferentiated CSC culture monolayers; (ii) the 3D maintenance of generated CSs in the differentiation medium implemented with BMP-4/β-FGF/activin A/Wnt-5a/Wnt-11 to induce a myogenic transcriptional program; and (iii) progressive cardiomyocyte specification within CSs using DKK1 treatment to inhibit the canonical Wnt/β-catenin pathway. Using this protocol, we first show that a 2D-to-3D switch guides/instructs myogenic plasticity within CSC-derived CSs. The 3D culture of CSs derived from CSCs in myogenic differentiation media results in the upregulation of cardiac troponin expression. When CSs are transferred back to a 2D condition on an ECM coating, approximately 70% of the cardiomyocytes within the CSs exhibit positive expression of troponin. Moreover, CS-iCMs upregulate the genes involved in calcium handling, indicating a progressive structural iCM maturation within the CSs.

To obtain a more efficient yield of cardiomyocytes at the end of the myogenic induction protocol, we explored different scaffold-free systems to generate CSs that are able to promote CSC myogenic differentiation. We show that cell density plays a crucial role in generating CSs with optimal sizes. Additionally, it enables the diffusion of oxygen, metabolites, and growth factors from the surface to the spheroid core center inducing a better iCM maturation. Indeed, we demonstrated that the number of cells seeded and the CS size modulate the expression of Tnnt2, Actc1, Myl2, and Myl7. While providing evidence that 3D cell aggregation per se guides the cardiomyocyte differentiation of CSCs, we also show that CS dissociation, as well as their subsequential reaggregation, boosts iCM differentiation, which could be considered an effective way of increasing CM specification and cardiac muscle representation within the CS. This phenomenon could be attributed to the ability of dissociation and reaggregation processes, which enable cells to mix and redistribute themselves, potentially in different states of differentiation, within the CSs. This reformation of homogeneous spheroids enhances the efficiency of myogenic differentiation. Similarly, during embryo development in vivo, cells affect one another in a spatiotemporal manner and within their microenvironment. It should be noted that the reaggregation step was tested using the ultra-low attachment culture condition with the 2D-to-3D switch. It would therefore be interesting to verify whether the re-aggregation step would even further maximize the differentiation and maturation of CSC-derived iCMs in the entirely 3D CS generation and myogenic differentiation protocol using agarose micro-molds.

Forced CSC aggregation to generate CSs and develop the myogenic protocol entirely in a 3D environment using agarose micro-molds maximizes iCM yield, lending further support to the essential role of 3D culture to obtain more developed cardiac muscle tissue in vitro from endogenous cardiac regenerative cells. Of interest, it has been previously reported that mesenchymal stem cells, endothelial progenitor cells, and c-Kit+ cardiac progenitor cells, when cultured together, spontaneously form scaffold-free 3D microenvironments, termed CardioClusters [53]. CardioCluster intramyocardial delivery improves cell retention and capillary density with the preservation of cardiomyocyte-size and long-term cardiac function in a murine infarction model [53]. Concurrently, it will be of interest to establish whether a scaffold-free 3D environment favorably impacts transcriptome diversity and population heterogeneity, as a molecular basis of the improved myogenic capacity in such condition [54]. Moreover, it remains to be tested whether a scaffold-based 3D environment can further improve CS viability, growth, and differentiation by providing an environment that is architecturally and functionally closer to the natural tissue [55]. Equally, it would be interesting to incorporate in silico/computational simulations, which may help in establishing better CS generation and differentiation protocols, as shown for other cell systems [56].

It should be highlighted that most of the evidence on the importance of 3D spheroids in mimicking in vivo cardiac conditions originates from the use of human embryonic and induced pluripotent stem cells (human pluripotent stem cells [hPSCs]) and their derivatives [20]. The current hPSC differentiation protocols yield cardiomyocytes that display an immature phenotype closely resembling the fetal stage of cardiomyocyte formation [57,58]. Several approaches have been implemented to overcome this developmental block and further mature the hPSC-derived cardiomyocytes [20,38]. It is therefore clear that our data from the present study would benefit from a direct comparison with the myogenic commitment of cardiac spheroids generated by mouse PSCs as a reference to clarify how mature iCMs from CSCs are when compared to iCMs from PSCs. Furthermore, it remains to be tested whether human CSCs have the same efficiency in CS generation and myogenic commitment as the mouse counterpart presented in this study. Finally, throughout the manuscript, we used the terms “differentiation” and “commitment” somewhat interchangeably. This was due to the need to describe the differentiation media and steps with the differentiated, and actually myogenic-committed, progeny. The issue is not trivial and should be taken into account as “differentiation” implies the acquisition of a wide range of phenotypic and functional characteristics, whereas “commitment” refers to lineage specification where only selective characteristics are evident.

In conclusion, a 3D culture system to generate CSC-derived CSs is instrumental for the highly efficient generation of iCMs from CSCs. Our results provide a novel approach to maximizing the production of iCMs from CSC-derived CSs using an entirely 3D environment, as offered by agarose micro-molds that will be useful in defining novel cardio-regenerative strategies and in potentially offering a novel approach for cell therapy in cardiovascular diseases.

## Figures and Tables

**Table 1:** List of antibodies used in the study and their specific application.

Antigen	Company	Application
C-kit	Miltenyi Biotec	FC
Sca-1	Miltenyi Biotec	FC
CD45	Miltenyi Biotec	FC
CD31	Miltenyi Biotec	FC
CD140 α	Santa Cruz Biotech	FC
CD34	Santa Cruz Biotech	FC
CD44	Miltenyi Biotec	FC
CD63	Miltenyi Biotec	FC
CD90	Miltenyi Biotec	FC
CD105	Miltenyi Biotec	FC
CD13	Santa Cruz Biotech	FC
CD166/Alcam	Miltenyi Biotec	FC
CD309	Miltenyi Biotec	FC
ROR2	Santa Cruz Biotech	FC
CD140 β	Miltenyi Biotec	FC
cTnI	Abcam	IF
Hif-1α	Abcam	IF
Ki67	Dako	IF
Connexin43	Cell Signaling	IF
CD45 microbeads, mouse	Miltenyi Biotec	Mouse CSC isolation
CD117 microbeads, mouse	Miltenyi Biotec	Mouse CSC isolation
CD31 microbeads, mouse	Miltenyi Biotec	Mouse CSC isolation

IF denotes immunofluorescence; FC denotes flow cytometry.

**Table 2:** List of RT-PCR primers.

Gene	ID Number
Gapdh	Mm99999915_g1
Nkx2.5	Mm01309813_s1
Gata4	Mm03053570_s1
Myh6	Mm00440359_m1
Myh7	Mm01319006_g1
Actc1	Mm01333821_m1
Tnnt2	Mm00441922_m1
c-kit	Mm00445212_m1
Atp2a	Mm01201431_m1
Pln	Mm00452263_m1
Hand1	Mm00433931_m1
Hand2	Mm00439247_m1
Connexin43	Mm01179639_s1
Ryr2	Mm00465877_m1
Mesp1	Mm00801883_g1
Mef2c	Mm01340842_m1
Nanog	Mm02384862_g1
Oct4	Mm03053917_g1
Myl2	Mm00440384_m1
Myl7	Mm00491655_m1
Ncx	Mm01232254_m1

## Data Availability

All data are available within this article.

## Supplementary Materials

The following supporting information can be downloaded at: https://www.mdpi.com/article/10.3390/cells12131793/s1, Figure S1: Cardiac transcription factors and pluripotency genes expression in response to 2D to 3D culture switch. (A) Bar graphs showing the cumulative qRT-PCR of cardiac transcription factors and contractile genes in differentiating WT-CSC, mT/mG-CSC and TomatoCSC at D0, D4, D8 and D14 of the myogenic commitment (n = 3 for each clone). Data are expressed as mean ± S.D.; Supplementary Table S1.
