# Supplementary material for: Adult Multipotent Cardiac Progenitor-Derived Spheroids: A Reproducible Model of In Vitro Cardiomyocyte Commitment and Specification
Source: Cells. 2023 Jul 5;12(13):1793. doi: 10.3390/cells12131793 (PMC10341123; doi:10.3390/cells12131793)
Supplement: Supplementary file 1 [file cells-12-01793-s001.zip › cells-2407492-supplementary.pdf]

cells

MDPI

Article

Differentiation of Cardiac stem cells derived spheroids: a reproducible model of in vitro cardiomyocyte commitment and specification

Mariangela Scalise, Fabiola Marino, Luca Salerno, Nunzia Amato, Claudia Quercia, Chiara Siracusa, Loredana Pagano, Andrea Filardo, Antonio Chiefalo, Giuseppe Misdea, Nadia Salerno, Antonella De Angelis, Konrad Urbanek, Daniele Torella, Eleonora Cianflone

Supplementary Material

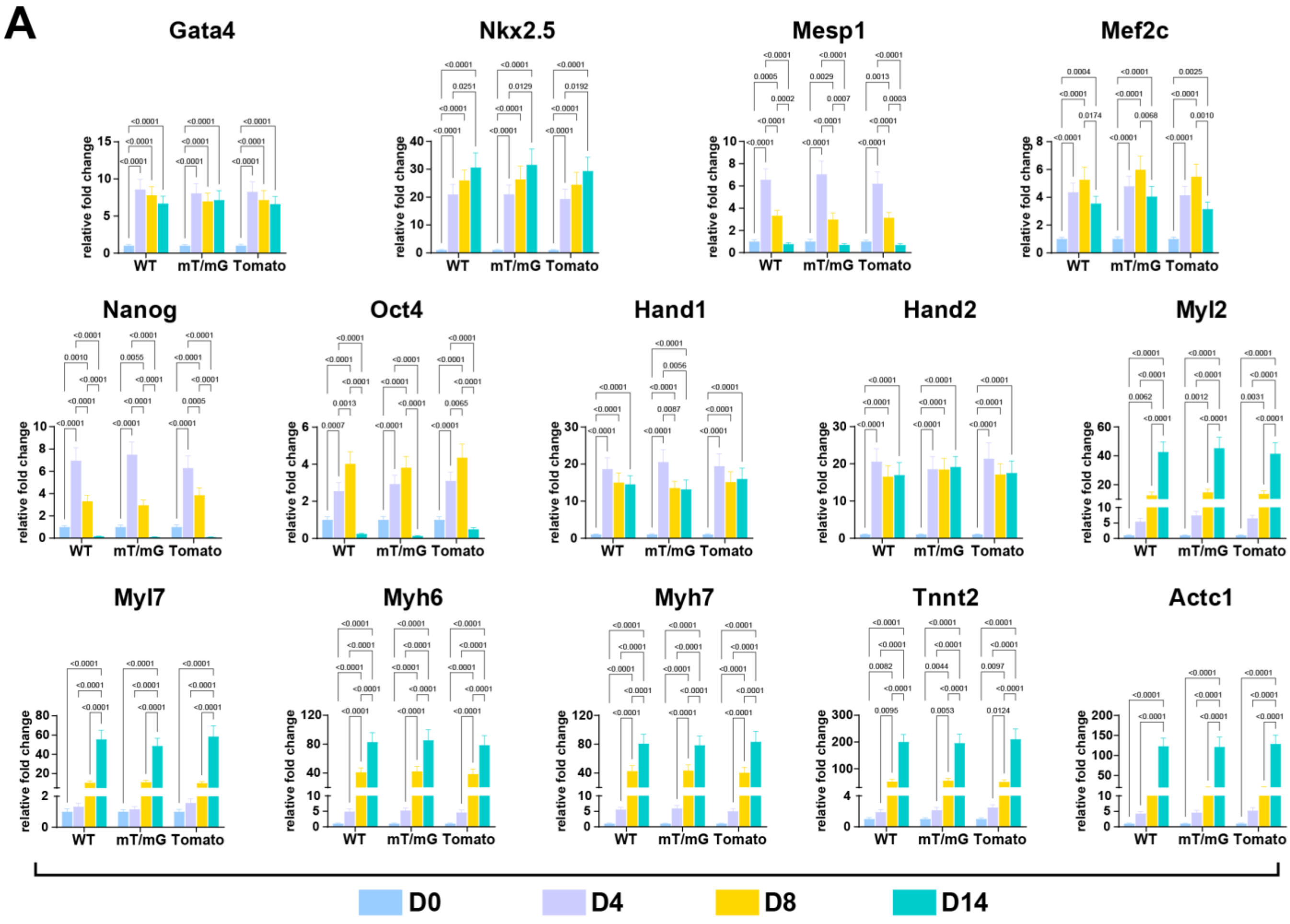

Supplementary Figure S1. Cardiac transcription factors and pluripotency genes expression in response to 2D to 3D culture switch. (A) Bar graphs showing the cumulative qRT-PCR of cardiac transcription factors and contractile genes in differentiating WT-CSC, mT/mG-CSC and Tomato-CSC at D0, D4, D8 and D14 of the myogenic commitment (n=3 for each clone). Data are expressed as mean ± S.D.

Supplementary Table S1

WT-CSCs

Marker	D0	D4	D8	D14
Sca-1	99,5±1%	56±2,4%	38,5±2,5%	99±0,5%
CD45	0,6±0,1%	0%	0%	0%
CD31	1,3±0,2%	0,5±0,1%	0,8±0,1%	1,1±0,2%
CD140α	98±2%	0,4±0,1%	2,5±0,3%	0,5±0,1%
CD34	3,8±0,1%	2,7±0,3%	0,1±0,1%	2,4±0,3%
CD44	95,6±3%	97,3±2,5%	85,6±5,5%	95,7±3%
CD63	98,6±2%	2,5±0,3%	36,9±1,5%	21,8±2,2%
CD90	0,1±0,1%	1,7±0,2%	0%	0%
CD105	99,6±2,5%	66,7±2,2%	10±0,9%	3±0,2%
CD13	2,7±0,3%	4,5±0,4%	2,1±0,3%	0,5±0,2%
CD166	84,6±4%	86±5%	99±1%	84±4%
CD309	3,3±0,3%	1,6±0,3%	3,4±1,3%	0,7±0,1%
ROR2	2,9±0,3%	1,8±0,2%	1,5±0,1%	0,9±0,1%
CD140β	44,9±4,6%	0%	0%	0%

mT/mG-CSCs

Marker	D0	D4	D8	D14
Sca-1	99±1%	55±2%	36±3%	99±0,1%
CD45	0,7±0,1%	0%	0%	0%
CD31	1±0,1%	0,3±0,1%	0,2±0,1%	1±0,1%
CD140α	96±2%	0,5±0,1%	2,5±0,3%	0%
CD34	4±0,1%	3±0,2%	0,1±0,1%	3±0,5%
CD44	93±1%	96±2%	80±5%	93±2%
CD63	97±2%	2,5±0,3%	33±1%	25±2%
CD90	0,1±0,1%	2±0,1%	0%	0%
CD105	95±1%	65±2%	11±1%	15±0,2%
CD13	3±0,2%	5±0,5%	2,3±0,5%	0,3±0,1%
CD166	96±1%	80±4%	96±2%	84±3%
CD309	4±0,5%	2±0,4%	2,8±2%	0,5±0,1%
ROR2	3,4±0,2%	2±0,2%	1,7±0,1%	1±0,1%
CD140β	40±4%	0%	0%	0%

Tomato-CSCs

Marker	D0	D4	D8	D14
Sca-1	96±2%	50±2%	34±3	96±1
CD45	0,6±0,1%	0%	0%	0%
CD31	1,5±0,2%	0,5±0,1%	0,1±0,1%	2±0,2%
CD140 α	95±2%	1±0,1%	0,8±0,2%	0%
CD34	3±0,1%	2,5±0,1%	0,1±0,1%	3±0,4%
CD44	92±0,5%	97±2,3%	82±4%	94±3%
CD63	93±2%	3±0,3%	30±3%	23±1%
CD90	0,3±0,1%	1,5±0,2%	0%	%
CD105	96±2%	60±3%	9±1%	2±0,2%
CD13	3,2±0,1%	4,5±0,5%	2,5±0,5%	0,4±0,1%
CD166	94±1%	78±2%	94±2%	82±3%

CD309	4,5±0,1%	2,2±0,4%	3±1%	0,9±0,1%
ROR2	3,0±0,1%	2,5±0,4%	2±0,2%	1,3±0,2%
CD140 β	42±3%	0%	0%	0%
